# Editorial: Perspectives in clinical infectious diseases: 2024/2025

**DOI:** 10.3389/fcimb.2026.1864691

**Published:** 2026-05-13

**Authors:** Ricardo Oliveira, Lorena V. N. Oliveira, Sónia Silva, Joana Castro

**Affiliations:** 1 INIAV – National Institute for Agrarian and Veterinary Research, Vila do Conde, Portugal; 2 Department of Medicine, University of Massachusetts Chan Medical School, Worcester, MA, United States; 3 CEB – Centre of Biological Engineering, University of Minho, Braga, Portugal; 4 LABBELS – Associate Laboratory, Braga, Guimarães, Portugal

**Keywords:** antimicrobial resistance (AMR), diagnostics and clinical management, emerging and rare pathogens, host susceptibility, infectious disease epidemiology, one health, therapeutic strategies

## 1 Introduction

Clinical infectious diseases remain a significant global health challenge, fueled by the ongoing emergence and re-emergence of pathogens, the rising threat of antimicrobial resistance (AMR), and the increasing complexity of populations dynamics. The interplay between microbial adaptation, host susceptibility, environmental pressures and healthcare system dynamics has profoundly reshaped the infectious disease landscape, making continuous innovation in diagnostics, therapeutics and surveillance essential.

Over the past decade, breakthroughs in molecular biology, genomics, and data-driven approaches have reshaped how infectious diseases are detected, evaluated and managed. However, persistent challenges such as delayed diagnosis, limited treatment options for resistant pathogens and disparities in healthcare access continue to impact patient outcomes worldwide. These issues are further exacerbated by the burden of chronic comorbidities, ageing populations, and globalization.

The Research Topic *“Perspectives in Clinical Infectious Diseases: 2024/2025”* brings together 25 contributions that collectively provide an overview of the current state of the field while also highlighting innovative directions and unresolved challenges. The included studies span a wide range of infectious diseases and clinical contexts, incorporating epidemiological analyses, diagnostic evaluations, therapeutic assessments, mechanistic insights, and case-based observations.

This Research Topic’s integrative approach is a major strength, reflecting the inherently interdisciplinary nature of infectious disease research. Collectively, the articles emphasize the importance of combining clinical expertise with advances in microbiology, immunology, molecular diagnostics, and public health. This Research Topic highlights the importance of a comprehensive approach that effectively bridges the gap between scientific research and clinical practice.

Thus, the goal of this editorial is to summarize the key themes addressed in this Research Topic, focusing on the scientific and clinical contributions of the included articles, and to offer insights into future directions in the field. Together, these studies provide valuable perspectives on evolving challenges and opportunities, reinforcing the critical importance of continued innovation and collaboration to address the global burden of infectious diseases.

## 2 Overview of the Research Topic

The contributions included in this Research Topic collectively illustrate the rapid evolution and increasing complexity of clinical infectious diseases. Rather than representing isolated advances, these studies converge to reveal several overarching trends that are reshaping the field: the growing integration of precision diagnostics into clinical workflows, the persistent and escalating challenge of AMR, the critical influence of host factors on disease susceptibility and outcomes, and the expanding role of interdisciplinary and One Health approaches in understanding and controlling infectious diseases.

Within this conceptual framework, the contributions can be categorized into four complementary and interrelated domains that mirror these emerging priorities: (*i*) infectious disease epidemiology and host susceptibility, (*ii*) antimicrobial resistance and therapeutic strategies, *(iii*) advances in diagnostics and clinical management, and (*iv*) emerging pathogens, rare infections, and One Health perspectives.

### 2.1 Infectious disease epidemiology and host susceptibility

A substantial number of contributions focus on the epidemiology of infectious diseases and the role of host-related risk factors in shaping susceptibility to and outcomes of these diseases. Liu et al. highlighted the increased risk of latent tuberculosis among patients with type 2 diabetes, reinforcing the importance of considering associated risk factors in the population. Similarly, Li et al. discussed the diagnostic and therapeutic challenges of latent tuberculosis in children with rheumatic diseases, emphasizing the importance of proactive management in immunologically vulnerable groups.

The influence of host-related factors in infection outcomes is further explored in studies addressing viral and systemic infections. Wang et al. showed the association between fatty liver disease and an increased risk of liver failure in patients with acute hepatitis B, illustrating how metabolic comorbidities influence disease progression and should be considered in early risks assessment and clinical management. Sun et al. investigated the impact of human papillomavirus infections on male reproductive health, highlighting the negative effects on fertility and the increased risk of transmission to partners, as well as the broader systemic consequences of viral infections. These findings emphasize that infectious diseases should not be viewed independently but rather understood within the broader context of overall patient health, including metabolic, immunological, and reproductive systems.

In parallel, genetic susceptibility is addressed by Lv et al., who identify a novel loss-of-function variant in STAT1 associated with Mendelian susceptibility to mycobacterial disease. Hua et al. explored the HBV-miR-3 and cGAS-STING axis, identifying a novel immunomodulatory pathway that may represent a promising therapeutic target in chronic hepatitis B. These studies highlight the importance of host genetics in determining the risk of infection, disease progression, and clinical outcomes, and pave the way for a more personalized approach managing infectious diseases. However, the translation of these findings into routine clinical practice remains in its early stages.

Together, these contributions emphasize that infections should not be understood in isolation, but instead as the result of complex interactions among pathogens, host biology, and underlying comorbidities.

### 2.2 Antimicrobial resistance and therapeutic strategies

AMR emerges as a central issue across several studies, reflecting its global health relevance and the ongoing need for effective therapeutic strategies to address resistant infections. Wang et al. provided a comparative analysis of bloodstream infections caused by carbapenem-resistant *Klebsiella pneumoniae* and *Escherichia coli* in hematological patients. The authors addressed the severe clinical impact of resistant infections in immunocompromised populations while also highlighting potential therapeutic alternatives, such as tigecycline or ceftazidime-avibactam, which were associated with improved outcomes. Similarly, Bazanelli Prebianchi et al. characterized hospital-associated methicillin-resistant *Staphylococcus aureus* (MRSA) genotypes responsible for community-onset infections, demonstrating the convergence of hospital and community reservoirs. In addition, they observed that deep tissue involvement, rather than specific bacterial genotypes, was the main predictor of severe outcomes and mortality.

Beyond resistance patterns, several studies in this Research Topic address the critical need for therapeutic optimization. Song et al. examined the prevalence and clinical impact of timely carbapenem administration, emphasizing the importance of pharmacokinetic/pharmacodynamic (PK/PD) target fulfillment in improving treatment efficacy. Zhou et al. compared the effectiveness and safety of vancomycin and linezolid in treating central nervous system infections. This provided evidence to inform therapeutic decision-making and suggested that, given their comparable effectiveness, treatment choice should be guided by patient-specific factors.

Innovative and adjunctive therapeutic approaches are also highlighted in the context of other severe diseases. Sun et al. reported the successful use of blood purification therapy in a patient with severe falciparum malaria complicated by cytokine storm and multiorgan dysfunction syndrome, indicating the potential of adjunctive therapies in critical care settings.

Collectively, these findings underscore the growing threat posed by resistant pathogens, particularly among vulnerable populations, and reinforce the importance of antimicrobial stewardship, early appropriate therapy, and PK/PD-guided treatment strategies. At the same time, they reveal both progress and persistent limitations in current therapeutic approaches, including the continued reliance on existing antimicrobial agents, many of which are decades old.

### 2.3 Advances in diagnostics and clinical management

Another key focus of this Research Topic is the advancement of diagnostic methodologies and their integration into clinical practice. Studies highlight the limitations of conventional culture-based techniques and the increasing clinical value of molecular approaches, reflecting a broader shift toward precision diagnostics in infectious diseases.

Lin et al. and Gao et al. evaluated metagenomic next-generation sequencing (mNGS) in comparison with conventional culture methods for periprosthetic joint infection and severe pneumonia, respectively. These studies highlight the superior sensitivity and broader pathogen detection capabilities of mNGS, particularly in complex or culture-negative cases. Similarly, Miguélez Sánchez et al. assessed the diagnostic performance of broad-range PCR in bacterial peritonitis, reinforcing the value of molecular diagnostics in clinical microbiology. Importantly, these approaches not only enhance diagnostic yield but also reveal the polymicrobial nature of infections that may otherwise remain undetected.

In addition to advances in pathogen detection, several studies underline the expanding role of biomarkers in guiding clinical management and therapeutic decision-making. Chen et al. identified serum ferritin as a predictive low-cost biomarker for interferon therapy response in chronic hepatitis B, while Shi et al. evaluated serum apolipoprotein A1 as a prognostic marker in elderly patients with SARS-CoV-2 infection. Complementing these findings, Zhou et al. analyzed determinants of functional cure in interferon-treated chronic hepatitis B, demonstrating the prognostic value of HBsAg dynamics and clinical predictors in guiding therapeutic strategies. These biomarker-driven approaches, particularly in the context of viral infections, further emphasize the growing importance of measurable biological indicators in predicting disease progression and informing individualized care.

The integration of diagnostics into clinical decision-making emerges as a critical determinant of impact. Kramme et al. demonstrated that combining rapid diagnostic testing with immediate infection consultation significantly increased the proportion of patients receiving targeted antibiotic therapy in septic intensive care settings. These findings underscore that diagnostic innovation alone is insufficient unless it is effectively integrated into clinical workflows and supported by timely expertise.

Taken together, these contributions illustrate a paradigm shift toward precision medicine in infectious diseases, where diagnostics, biomarkers, and clinical decision-making are increasingly integrated to optimize outcomes and patient care. However, the implementation of precision diagnostics and biomarker-guided strategies in routine practice remains uneven. Barriers related to cost, accessibility, data interpretation, and variability in biomarker performance across populations continue to limit widespread adoption, highlighting the need for further validation and standardization.

### 2.4 Emerging and rare pathogens, and One Health perspectives

The emergence of novel and rare pathogens, together with evolving epidemiological patterns among well-established organisms, remains a critical challenge in clinical infectious diseases. Collectively, the contributions in this section underscore the importance of continuous surveillance, advanced diagnostics, and integrative approaches to better understand pathogen dynamics across different contexts.

Chen et al. provided a comprehensive analysis of candidemia over a three-year period, offering valuable epidemiological insights into fungal infections. In parallel with these findings, case reports by Lu et al. and Huang et al. describe infections caused by the fungal pathogen *Trichosporon asahii* and the rare bacterial species *Wohlfahrtiimonas*, respectively, underscoring the clinical relevance of uncommon and opportunistic pathogens, particularly in immunocompromised individuals. These studies illustrate the expanding spectrum of infectious agents encountered in modern clinical practice and the challenges associated with their diagnosis and management.

At the population level, Valenzuela et al. analyzed an emm1-dominant group A *Streptococcus* outbreak, situating it within a broader epidemiological context and demonstrating the importance of longitudinal surveillance in outbreak detection and characterization. Similarly, Liu et al. described the prevalence of multiple respiratory pathogens among children using multiplex PCR, highlighting the complexity of pathogen circulation in community settings and the value of comprehensive surveillance strategies.

A particularly important cross-cutting theme in this Research Topic is the adoption of One Health and genomic surveillance approaches. In this context, Gomes et al. emphasized the role of whole genome sequencing (WGS) in monitoring foodborne pathogens, aligning with the One Health framework. This approach aligns with the One Health framework, which recognizes the interconnectedness of human, animal, and environmental health, and underscores the need for interdisciplinary strategies in infectious disease control.

In addition, Akula et al. raised important considerations regarding laboratory practices and experimental integrity, demonstrating that methodological factors can significantly influence both infectious disease research and the interpretation of immunological findings.

Collectively, these studies highlight the need for robust surveillance systems, methodological rigor, and cross-sector collaboration. They further emphasize that addressing emerging and rare pathogens requires not only heightened clinical awareness but also integrated approaches that combine genomic technologies, epidemiological monitoring, and One Health principles.

## 3 Conclusions and future perspectives

The Research Topic *“Perspectives in Clinical Infectious Diseases: 2024/2025”* provides a comprehensive and timely synthesis of current advances, ongoing challenges, and emerging directions in the field of clinical infectious diseases. The 25 contributions included in this Research Topic taken together demonstrate that infectious diseases remain a highly dynamic and multifaceted domain, requiring continuous adaptation of diagnostic, therapeutic, and surveillance strategies.

A central message from this Editorial is the expanding role of precision diagnostics and molecular technologies in transforming clinical practice. Advances such as mNGS and broad-range molecular assays are enabling faster, more accurate, and more comprehensive pathogen detection, particularly in complex and polymicrobial infections. However, their full clinical impact depends on effective integration into healthcare systems, underscoring the need to bridge technological innovation with clinical expertise, infrastructure, and standardized workflows.

At the same time, the persistent global threat of AMR continues to challenge current treatment paradigms. The studies included in this Research Topic highlight the urgent need for strengthened antimicrobial stewardship, optimization of therapeutic strategies, and sustained investment in the development of new antimicrobial agents. The disproportionate burden of resistant infections in vulnerable populations further emphasizes the need for coordinated and globally aligned responses.

Another key insight is the growing recognition of the influence of host-related factors, including comorbidities, immune status, and genetic predisposition, on infectious disease susceptibility and outcomes. Integrating these factors into clinical decision-making supports the movement toward more personalized and patient-centered approaches to care.

Altogether, the contributions in this Topic highlight the importance of addressing both common and rare infections, as well as the continuous emergence of new pathogens and clinical scenarios. The inclusion of case reports, outbreak analyses, and epidemiological studies reflects the need for vigilance and adaptability in clinical practice, particularly in the context of immunocompromised patients and evolving microbial landscapes.

Importantly, this Research Topic also reinforces the value of interdisciplinary collaboration and One Health approaches, integrating human, animal, and environmental health perspectives. Such strategies are essential for understanding transmission dynamics, improving surveillance systems, and enhancing preparedness for future infectious disease threats.

Despite these advances, several limitations and gaps remain evident across the included studies. Many are retrospective in design, limiting causal inference, while others rely on single-center data, potentially restricting generalizability. In addition, the translation of advanced diagnostics and biomarker discoveries into routine clinical practice remains incomplete, often hindered by logistical, financial, and infrastructural barriers.

Addressing these challenges will require well-designed prospective, multicenter, and interventional studies, alongside sustained investment and international collaboration. More broadly, continued progress in clinical infectious diseases will depend not only on technological and scientific innovation but also on effective integration, translation, and equitable accessibility, supported by close coordination among clinicians, microbiologists and immunologists, epidemiologists, and public health professionals.

In conclusion, the contributions presented in this Research Topic reflect the current state of clinical infectious diseases and shed light on the path forward. By promoting interdisciplinary collaboration and embracing emerging technologies, the field is well-positioned to address the evolving challenges posed by infectious diseases and improve patient outcomes worldwide.

